# No elevated levels of orthorexic eating behavior in a sample of adults with allergies and food intolerances

**DOI:** 10.1007/s40519-022-01498-0

**Published:** 2022-10-21

**Authors:** Friederike Barthels, Leonie Bamberg, Reinhard Pietrowsky

**Affiliations:** Department of Clinical Psychology, Institute of Experimental Psychology, Heinrich Heine University Düsseldorf, Universitätsstraße 1, 40225 Düsseldorf, Germany

**Keywords:** Orthorexia nervosa, Orthorexic eating behavior, Allergic diseases, Food intolerances

## Abstract

**Purpose:**

To compare orthorexic eating behavior in a sample of adults with and without self-reported allergies and food intolerances.

**Methods:**

*N* = 52 individuals with and *n* = 102 individuals without self-reported allergies and food intolerances (80% with medical diagnosis; in total 74.6% female, 23.7% male; age: *M* = 28.13, *SD* = 11.96 years) completed an online survey with the Düsseldorf Orthorexia Scale and answered several questions regarding their allergies/intolerances.

**Results:**

The groups did not differ in their orthorexic eating behavior. In the sample of individuals with allergies/intolerances, orthorexic eating behavior correlated with the perceived severity of the allergic symptoms and the number of consequences that the allergies had for eating behavior.

**Conclusions:**

In line with previous findings, orthorexic eating behavior does not seem to be elevated in individuals with allergies/intolerances. However, focusing on a healthy diet despite adverse food reactions and experiencing a number of allergy-related consequences for one’s eating behavior might be associated with orthorexic eating behavior.

**Level of evidence:**

III, case–control analytic study.

## Introduction

Individuals with allergies, whether they have food intolerances, food allergies or cross-sensitivities due to other, non-food allergies, need to pay close attention to their eating behavior. Depending on the severity of the adverse food reactions, the potential allergens have to be avoided to a varying degree of strictness, otherwise leading to a variety of symptoms, e.g., oral or gastrointestinal issues up to and including a life-threatening anaphylaxis [1]. Interestingly, the prevalence of diagnosed food allergies in adults in Western countries is about 2%, whereas a much larger number of individuals believes to have adverse food reactions [1]. The (in some cases: belief of) necessity to avoid allergens leads to several consequences in eating behavior, e.g., reading the list of ingredients thoroughly to identify allergens, refusal to taste new food or food with unknown ingredients and constantly thinking about food/allergens and potential consequences when accidentally consuming them. It also likely that these consequences are subject to various influencing factors, such as symptom severity, and that there may be differences in behavior between individuals who actually have a diagnosed allergy compared to those who only believe to have one.

The aforementioned consequences resemble orthorexic behavior, which is defined as a fixation on only eating food considered healthy [2]. According to [2], diagnostic criteria proposed so far include (1) obsessional preoccupation with healthy eating, (2) emotional consequences (e.g., anxiety) and (3) psychosocial and physical impairments. Both individuals with orthorexia and individuals with allergies/intolerances avoid foods according to specific criteria, hence, restrict their diet, and might experience distress and fear when being confronted with unknown food. Furthermore, Bratman [3], who coined the term orthorexia nervosa, described a few cases, where adverse food reactions and orthorexic eating behavior were intertwined. Starting from the medical necessity to avoid certain foods, some individuals eventually excluded more and more foods from their diet believing that they might cause them harm, too.

However, studies investigating the relation between orthorexic eating behavior and allergies/intolerances have yielded controversial results. In one study, individuals with one or more food intolerances displayed higher levels of orthorexic eating behavior [4], whereas in two other studies, this was not the case [5, 6]. Since the aforementioned studies were not specifically designed to investigate orthorexic eating behavior in the context of allergies and intolerances, they did not assess these conditions in detail, maybe missing important influencing factors.

Hence, the aim of this study was to investigate the relation between orthorexic eating behavior and allergies/intolerances while also taking into account potential moderating factors, such as the number of allergies/intolerances, the number of symptoms, the number of consequences that the allergies/intolerances have for the eating behavior, the perceived subjective distress due to the consequences, the perceived influence of allergies on eating behavior, the frequency of avoidance of foods due to adverse food reactions, and the perceived severity of the symptoms. We hypothesized that individuals with allergies/intolerances display higher levels of orthorexic eating behavior. Furthermore, we assumed that the aforementioned characteristics of the allergies/intolerances might be associated with orthorexic eating behavior as well.

## Methods

### Sample

Using an online questionnaire, data were collected anonymously without recording IP addresses. Participants were recruited via social networks. They were informed that their participation was voluntary and anonymous and that their data were handled according to privacy policy. Furthermore, participants were informed that they could cancel the survey at any time by not completing or by not submitting the questionnaire. By sending their data and by ticking a box with the statement “I agree to participate in the study”, informed consent was obtained. In total, 233 data sets were recorded. 60 data sets were removed (duplicates: *n* = 19, missing informed consent: *n* = 34, missing data: *n* = 7), so the remaining sample consisted of 173 participants. Legal age of 18, providing informed consent, clear indication regarding allergy/no allergy and completing the questionnaire served as inclusion criteria.

*N* = 129 females, *n* = 41 males, *n* = 1 other and *n* = 2 participants who did not report their gender (age: *M* = 28.13, *SD* = 11.96 years, BMI: *M* = 22.84, *SD* = 3.36 kg/m^2^; 63.6% students, 28.9% employed; 71.1% with qualification for university entrance, 17.9% high school graduates) participated in the study. *N* = 12 participants without current allergies/intolerances were excluded, because they reported to have experienced allergies/intolerances in the past and *n* = 7 individuals were excluded, because they were not sure whether they currently had any allergy/intolerance. The resulting groups with allergies/intolerances (*n* = 52, abbreviated AG) and without (*n* = 102, abbreviated CG) did not differ regarding age (*M*_AG_ = 30.27, *SD*_AG_ = 12.83 years vs. *M*_CG_ = 26.92, *SD*_CG_ = 11.30 years; *t*(92.03) =  − 1.593 *p* = 0.115), BMI (*M*_AG_ = 23.02, *SD*_AG_ = 3.80 kg/m^2^ vs. *M*_CG_ = 22.50, *SD*_CG_ = 3.19 kg/m^2^; *t*(86.95) =  − 0.822, *p* = 0.413) nor gender (AG: 76.9% female, 21.2% male, 1.9% missing; CG: 71.6% female, 26.5% male, 1.0% other, 1.0% missing; *χ*^2^(3) = 1.275, *p*_asymptotic_ = 0.735).

With 75% (*n* = 39), the majority of the AG reported allergies to both foods and other substances. 25% (*n* = 13) reported only allergies to other substances (e.g., pollen, house dust mites, animal hair), with one participant (7.7%) reporting cross-sensitivities and another participant (7.7%) being not sure regarding cross-sensitivities. Since cross-sensitivities/cross-reactions to foods are highly prevalent in individuals with non-food allergies, especially in those allergic to pollen [for an overview, see 7], we decided to not exclude these participants, because it is likely that they nonetheless observe or change their eating behavior due to the possibility of experiencing cross-reactions. 80% (*n* = 42) of the AG reported to have received a medical diagnosis for their allergies, 15.4% (*n* = 8) reported to not have received a medical diagnosis and 3.8% (*n* = 2) were unsure.

Reported occurrences of allergic symptoms were skin issues (67.3%, *n* = 35 of the participants), gastrointestinal problems (44.2%, *n* = 23), nausea (21.2%, *n* = 11), mucosal reactions (51.9%, *n* = 27, sneezing and rhinitis (59.6%, *n* = 31) and dyspnea (38.5%, *n* = 20).

Reported occurrences of consequences that the allergies had on the participant’s eating behavior were avoidance of social events (5.8%, *n* = 3 of the participants), bringing one’s own food to work or meetings with friends and family (23.1%, *n* = 12), accurate reading of the list of ingredients (46.2%, *n* = 24), fear of accidental allergen ingestion (19.2%, *n* = 10), unbalanced eating behavior due to multiple allergies (13.5%, *n* = 7), exclusive consumption of self-prepared food (5.8%, *n* = 3) and actively trying to eat extra healthy (34.6%, *n* = 18).

### Measures

To assess orthorexic eating behavior, the Düsseldorf Orthorexia Scale (DOS) [8] was used. It consists of 10 items to be rated on a 4-point scale ranging from *does not apply to me* (1) to *applies to me* (4), with high scores indicating high levels of orthorexic eating behavior. In this sample, Cronbach’s alpha was = 0.854.

Using self-designed questions, the following aspects regarding allergies/intolerances were surveyed: the presence and number of allergies/intolerances and if they had been medically diagnosed, the presence of cross-sensitivities, past experiences with allergies/intolerances, indication of what the participants were allergic/intolerant to, number of symptoms (e.g., oral and gastrointestinal issues) and perceived severity of symptoms [5-point scale from *barely present* (1) to *very severe* (5)], influence of the allergies/intolerances on eating behavior [5-point scale from *not at all* (1) to *very much* (5)], frequency of avoidance of foods due to adverse food reactions [5-point-scale from *never* (1) to *always* (5)], number of consequences that the allergies have for the eating behavior (e.g., checking ingredients, avoiding social events etc.) and subjective distress perceived due to these consequences [5-point scale from *not at all* (1) to *very much* (5)].

### Design and analysis

All analyses were conducted with IBM SPSS Statistics 28 for Mac OS. The sum score for the DOS was calculated according to the information in the corresponding publication. To calculate the number of allergies with respect to the number of affected substances, allergies to single substances (e.g., eggs, apples) counted once, allergies to groups of substances (e.g., nuts, pollen) counted twice and intolerances (e.g., gluten, fructose, lactose, histamine) counted threefold.

Regarding descriptive data, means (*M*), standard deviations (*SD*), absolute and relative frequencies are reported. An independent *t* test was used to compare the mean score of the DOS between the AG and the CG. For additional exploratory sub group analyses, due to small samples sizes, the non-parametric Kruskal–Wallis test was used. In the AG sub sample, Pearson correlations were computed between DOS scores and characteristics of the allergies/intolerances. For all analyses, an alpha level of 0.05 was used. Sample sizes may vary due to missing values.

## Results

### Group comparisons

No significant differences were observed in DOS scores between the AG and CG (*M*_AG_ = 17.33, *SD*_AG_ = 4.56 vs. *M*_CG_ = 17.97, *SD*_CG_ = 5.84, *t*(151) = 0.693, *p*_one-tailed_ = 0.245).

For an exploratory analysis, *n* = 13 individuals who reported to only have allergies to non-food substances were compared to *n* = 39 individuals who reported to have both food and other allergies, and to the CG without allergies (*n* = 101). No significant differences in the DOS scores were found between these three groups (*M*_food+other_ = 17.45, *SD*_food+other_ = 4.54 vs. *M*_non-food_ = 17.00, *SD*_non-food_ = 4.80 vs. *M*_CG_ = 17.97, *SD*_CG_ = 5.84; *H*(2) = 0.058, *p*_asymp,two-tailed_ = 0.971).

### Correlations

In the AG sub sample, the number of consequences that the allergies had for the eating behavior (*r* = 0.448, *p* < 0.001) and the perceived severity of the allergic symptoms (*r* = 0.307, *p* = 0.027) correlated with the DOS (all other *p* > 0.05).

## Discussion

In contrast to our hypothesis, individuals with allergies and intolerances did not display higher levels of orthorexic eating behavior. Hence, the presence of allergies/intolerances does not seem to be accompanied by a fixation on healthy eating, suggesting that restricting one’s eating behavior due to a medical necessity is not associated with further restrictions in terms of healthy eating.

Regarding moderating factors, the perceived severity of allergic symptoms correlated with orthorexic eating behavior. It could be hypothesized that focusing on a healthy diet might be accompanied by eating foods considered healthy despite perceiving adverse food reactions. Furthermore, the number of consequences that the allergies had for the eating behavior correlated with orthorexic eating behavior. The consequences queried contained mostly avoidance behaviors, e.g., only eating self-prepared food, checking ingredients and avoiding social events, which resemble the orthorexic avoidance behavior assessed with the DOS. Taking into account the other results which do not suggest that allergy-related restrictions are associated with orthorexic eating behavior, it could be supposed that respecting necessary consequences is accompanied by perceiving one’s diet as healthier and hence, by scoring higher in the DOS.

Taking a look at the present study against the background of the current state of research, it becomes apparent that there are now—including this survey—three studies [5, 6] reporting no relationship between orthorexia nervosa and allergies and one study [4] that found higher levels of orthorexic eating behavior in individuals with one or more allergies. However, since potential influencing factors (e.g., number of allergies/intolerances and symptoms, consequences on eating behavior) have not been investigated in other studies, these results and the aforementioned interpretations should be verified in future research.

### Strengths and limitations

This is the first study specifically investigating a group of adults with allergies/intolerances and assessing these conditions in detail, to find potential influencing factors. However, this study relies on self-reports of allergies and food intolerances, hence, a verification using medical tests and diagnoses was not conducted, which might influence the validity of the results. This is especially true for 20% of the participants who do not have a medical diagnosis or are uncertain about it. Hence, whether an allergy/intolerance or other medical factors cause the symptoms remains unclear. In addition, it was not assessed since when the individuals experience allergic symptoms. There might be differences between individuals who are accustomed to their allergy/intolerance and those newly diagnosed in terms of coping strategies. Furthermore, the recruited convenience sample is rather young and highly educated, and might, therefore, not be representative of individuals with allergies and intolerances in general.

## Conclusions

This brief report supports the results of previous studies suggesting that individuals with allergies and intolerances do not display higher levels of orthorexic eating behavior. This could indicate that orthorexic eating behavior is rather a mental disorder than a reaction to medically necessary restrictions in eating behavior. Furthermore, the results suggest a qualitative difference between a pathological preoccupation with food quality and being concerned about eating foods that one knows are potentially harmful to one’s health. Future studies should verify these results, especially regarding potential influencing factors (e.g., by also assessing since when the individuals experience allergic symptoms), in a sample of participants with a verified medical diagnosis of allergies and intolerances.

### What is already known on this subject?

While two studies suggest that orthorexic eating behavior is not associated with allergies/intolerances, one study reveals a relation between these two aspects, with no study having specifically investigated a group of adults with allergies/intolerances and potential influencing factors in detail.

### What does this study add?

In line with two previous studies, this study did not find elevated levels of orthorexic eating behavior in a sample of individuals with allergies/intolerances, suggesting that restricting one’s eating behavior due to a medical necessity is not associated with further restrictions in terms of healthy eating. However, the severity of allergic symptoms and the number of consequences that the allergies/intolerance have for the eating behavior might be influencing factors.

## Data Availability

Upon request, original data are available from the corresponding author.
